# Experimental Investigation of Ultra-High Molecular Weight Polyethylene Fibers and Fabric for Flexural Reinforcement in Ultra-High-Performance Concrete

**DOI:** 10.3390/ma18092002

**Published:** 2025-04-28

**Authors:** Zengrui Pan, Faning Dang, Rabin Tuladhar, Shi Yin, Feng Shi, Peter To, Zisheng Tang

**Affiliations:** 1 State Key Laboratory of Eco-hydraulics in Northwest Arid Region of China, Xi’an University of Technology, Xi’an 710048, China; zengrui.pan@my.jcu.edu.au (Z.P.); dangfn@mail.xaut.edu.cn (F.D.); 2 College of Science and Engineering, James Cook University, Townsville, QLD 4811, Australia; peter.to@jcu.edu.au (P.T.); albert.tang@jcu.edu.au (Z.T.); 3 School of Engineering and Technology, Central Queensland University, Rockhampton, QLD 4701, Australia; 4 Ningbo Shike New Material Technology Co., Ltd., Ningbo 315000, China

**Keywords:** UHMWPE, UHPC, textile reinforced mortar, fiber reinforced polymer, hybrid reinforcement, flexural behavior

## Abstract

This study investigates the use of Ultra-High Molecular Weight Polyethylene (UHMWPE) fibers and fabric to enhance the flexural performance of Ultra-High-Performance Concrete (UHPC). A total of 45 specimens were tested to examine the effects of fiber type, fabric material, adhesive, and various combined strengthening techniques. The main findings are that incorporating UHMWPE fiber into the ultra-high-strength mortar (HSM) matrix provides superior performance compared to steel fiber, particularly in enhancing crack resistance and energy absorption. UHMWPE fiber-reinforced UHPC achieved a flexural toughness of 307 KJ/m^3^, over three times higher than that of steel fiber-reinforced UHPC (98 KJ/m^3^). The use of UHMWPE fabrics was more effective in improving the ductility and toughness of the composites than the use of glass fabrics. The bonding effect of using epoxy resin with UHMWPE fabric is better than using magnesium phosphate cement (MPC). Increasing the number of fabric layers improved the flexural properties of externally bonded fabric but had no impact on internal reinforcement techniques. The best strengthening method in this study was a combination of incorporating UHMWPE fiber internally and externally bonded fabric on a concrete surface, yielding the highest toughness of 580 KJ/m^3^.

## 1. Introduction

UHPC is a cement-based composite material characterized by ultra-high strength, high toughness [1], excellent durability [2,3], high packing density [4], and high internal uniformity [5]. UHPC typically has a compressive strength exceeding 150 MPa, a tensile strength exceeding 15 MPa, and a packing density above 0.90 [6,7,8,9]. Its flexural toughness is often reported to exceed 90–120 KJ/m^3^ [10]. UHPC exhibits excellent durability, with chloride ion permeability below 40 Coulombs and relative dynamic modulus retention over 96% after extended freeze–thaw cycles [11]. Given the same design strength, the design dimensions for UHPC can be less than half of those for ordinary concrete, which makes it a preferred material for bridge decks, building facades, high-rise structures, and other high-stress applications. Its excellent mechanical properties and resistance to environmental factors make UHPC highly desirable for infrastructure projects [12]. However, the inherent brittleness and low tensile strength of unreinforced HSM limit its use in bending structures, necessitating reinforcement materials to achieve the required performance levels [13].

Traditional reinforcement methods for UHPC often involve the addition of steel fibers [12], providing enhanced tensile strength and improved flexural behavior while decelerating the propagation of cracks [14,15]. While these reinforcement materials improve the structural performance of UHPC, they also present certain limitations. Steel fibers, for instance, add significant weight to concrete and are prone to corrosion, particularly in environments with high moisture or corrosive chemicals. The construction of steel fiber would hurt the fingers of workers. Crack formation in the tensile zone of a flexural member exposes steel fibers to moisture in the air, leading to a significant reduction in durability. With the addition of steel fiber and other admixtures, the price of UHPC can be as high as 2500–3000 USD/m^3^, significantly higher than ordinary concrete at the rate of around 170 USD/m^3^ [16]. These disadvantages have prompted the exploration of alternative reinforcement materials that can maintain or enhance the desired properties of UHPC without these disadvantages.

UHMWPE has emerged as a promising alternative to traditional steel reinforcement in UHPC [17,18]. This advanced polymer offers several beneficial properties, including a higher tensile strength of 3700 MPa compared to 500 MPa for steel [19,20], a lower density of 970 kg/m^3^ compared to 7840 kg/m^3^ for steel [20,21], and exceptional resistance to corrosion and abrasion [22,23]. Due to its overwhelmingly higher strength-to-weight ratio, UHMWPE fiber offers significant economic advantages compared to steel fiber. For example, with a 2% volume fraction of reinforcing fiber in the concrete matrix, the cost of incorporating UHMWPE fiber is approximately 40 USD/m^3^, much lower than the 95 USD/m^3^ required for steel fiber [20,24]. These characteristics make UHMWPE an ideal candidate for reinforcing UHPC, particularly in applications where weight and durability are critical factors.

Despite its advantages, UHMWPE has been relatively underexplored in the context of UHPC-reinforced flexural behavior. Conventional reinforcement materials, like steel fibers, while effective, have drawbacks, such as potential corrosion and increased weight. The unique properties of UHMWPE, such as its light weight and resistance to environmental degradation, present a compelling case for its use in UHPC reinforcement [25]. However, further investigation is needed to understand how UHMWPE fibers and other UHMWPE-based materials perform when integrated into UHPC, especially in bending structures.

The current reinforcement method for UHPC faces several challenges. Steel fibers, as mentioned earlier, are prone to corrosion and add extra weight. Due to the low water-to-cement ratio and the addition of macro metallic fibers, the reduced workability of UHPC makes casting difficult [16,26]. This has prompted the exploration of alternative reinforcement methods that address these limitations while offering comparable or superior performance.

The use of textiles or fiber-reinforced polymers (FRPs) has been proposed to replace conventional steel reinforcement due to their low density (typically 1.2–2.0 g/cm^3^) and excellent corrosion resistance, especially in aggressive environments where steel is prone to chloride-induced corrosion [27,28]. For example, embedding mesh-shaped fabric into the UHPC matrix enhances its flexural behavior [29]. Additionally, externally bonded FRPs can also improve load-bearing capacity and durability [30,31]. Commonly used FRPs are made from carbon or glass [29,31]. However, UHMWPE fiber has a lower density, higher strength, and greater elongation than carbon or glass fibers [21,32].

Currently, the most commercially available adhesives for externally bonded FRPs on concrete are based on epoxy resin, and they have better mechanical properties than other thermosetting adhesives [33]. However, Chen et al. [34] found out that some fillers of resin are toxic heavy metals that cause harmful effects on health and the environment. To improve those weaknesses, researchers have explored MPC as an alternative green and economical repair material. According to Kejia [35], MPC can create a good bonding result in the repair of damaged concrete structures.

The use of concrete reinforced with hybrid fiber and fabric is also an innovative technology. Meszoly et al. [36] investigated the effects of combined fiber and textile reinforcement on the flexural behavior of UHPC slabs. Specifically, the reinforcement of high-performance fabric, working in tandem with the steel fibers inside UHPC, can further improve the bending resistance of the specimens [37]. Meng et al. [29] demonstrated that textile-reinforced UHPC achieves higher toughness than normal UHPC and greater strength than textile-reinforced mortars. Furthermore, externally bonding UHPC with FRPs is another pathway for improving the mechanical behavior of UHPC [38,39,40]. UHPC resists compressive stress, while FRPs provide high tensile resistance, ranging from 600 MPa to over 2000 MPa, depending on the type of fiber used [41]. FRP wrapping on the tensile face of bending components significantly resists crack propagation [42].

In previous studies, most researchers focused on shifting fiber types within the matrix, such as incorporating hybrid steel and UHMWPE fibers [43,44] or replacing steel entirely with other synthetic fibers [45,46]. Additionally, the use of other fiber or fabric-strengthening technologies has also shown potential benefits. Meng et al. [29] investigated the effect of embedded and externally bonded CFRPs or GFRPs on the flexural behavior of the matrix combined with fiber incorporation. Fu et al. [47] compared the flexural performance of carbon, glass, and basalt mesh-shaped textiles embedded in UHPC. However, there is limited research on using combined UHMWPE fiber and fabric materials to strengthen the flexural properties of UHPC.

This research aims to investigate the strengthening effects of UHMWPE fiber and UHMWPE fabric on the flexural behavior of UHPC. The significance of this research lies in its potential to fill a critical gap in the literature and guide the development of more effective reinforcement techniques for UHPC in bending applications. The experimental materials were mainly divided into two categories: HSM and UHPC. HSM refers to the plain cementitious composite without any fiber or fabric reinforcement, while UHPC refers to HSM reinforced with fibers. Additionally, UHPC bonded with textiles internally, and FRPs externally were also investigated. This paper seeks to quantify the improvements in flexural strength, ductility, and toughness of these reinforcement methods. This comparative analysis will provide insights into how UHMWPE-based reinforcement methods can be optimized for bending structures.

The organization of the paper is as follows. Section 2 presents the materials and experimental methods. Section 3 discusses the test results and provides a detailed analysis of load–displacement behavior, flexural properties, cost–performance, and fracture mechanisms. Section 4 summarizes the key findings of this study. Section 5 outlines the limitations and proposes directions for future research.

## 2. Experimental Program

### 2.1. Materials

The UHPC used in this study is readily available in the market and was purchased from a local concrete manufacturer. Its main ingredients include cement, sand, fiber, and water, as shown in Table 1. The compressive strength of UHPC is 120 MPa.

**Figure 1:**
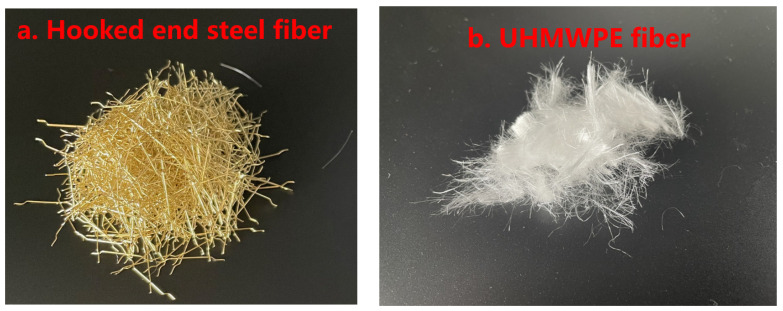
The two types of reinforced fibers used in this study: (**a**) hooked-end steel fibers and (**b**) UHMWPE fibers.

The fiber materials used in this study were steel fiber and UHMWPE fiber. The properties of the fibers are presented in Table 2. The hooked-end steel fibers were produced by Tai’an Tonghong Fiber Co., Ltd., China (Tai’an, China), as shown in Figure 1a. The UHMWPE fiber, BFL12, was produced by Ningbo Shike New Material Technology Co., Ltd., Ningbo, China, as shown in Figure 1b. The fiber volume fraction of UHMWPE and steel fibers are 2.0% and 1.2%, respectively, in the UHPC matrix, according to manufacturing suggestion. The dimensions of the concrete beam used in all the tests were 160 mm × 40 mm × 40 mm.

**Figure 2:**
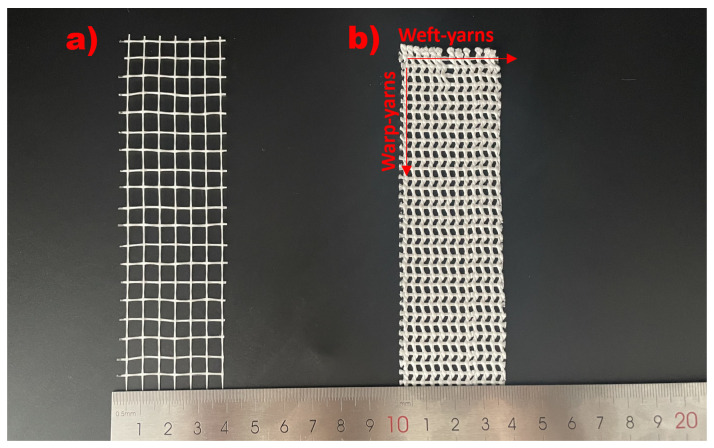
The two types of reinforced fibers used in this study: (**a**) glass mesh fabric and (**b**) UHMWPE mesh-shaped fabric.

**Figure A1:**
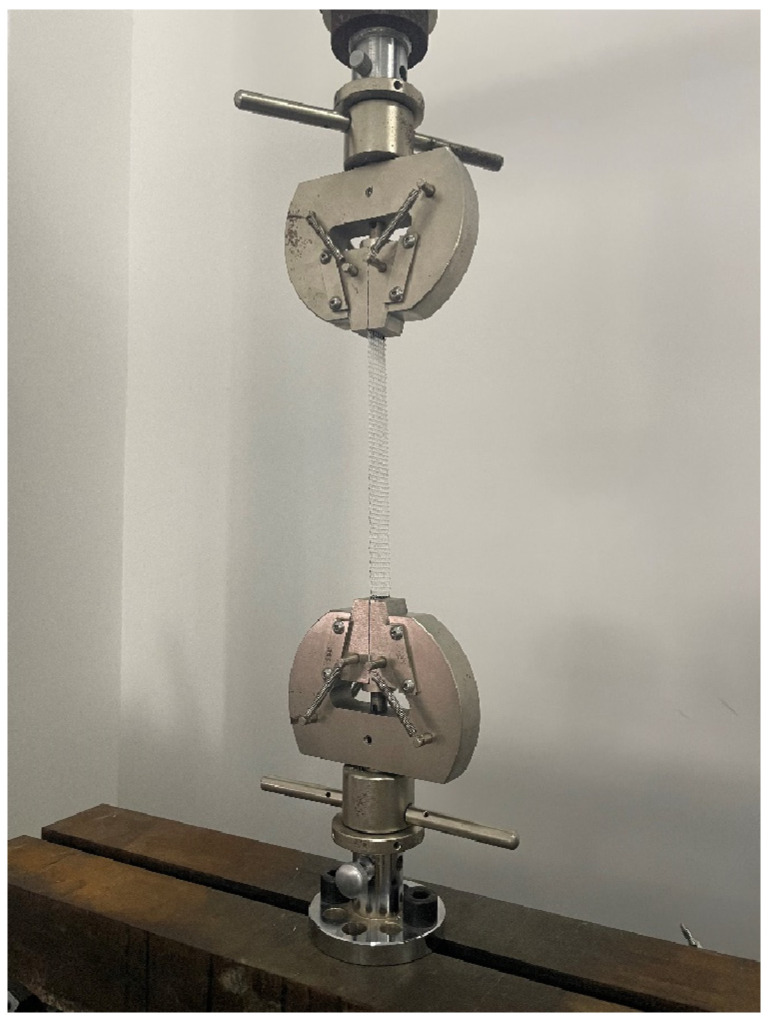
The test setup of the uniaxial tensile test for fabric.

**Figure A2:**
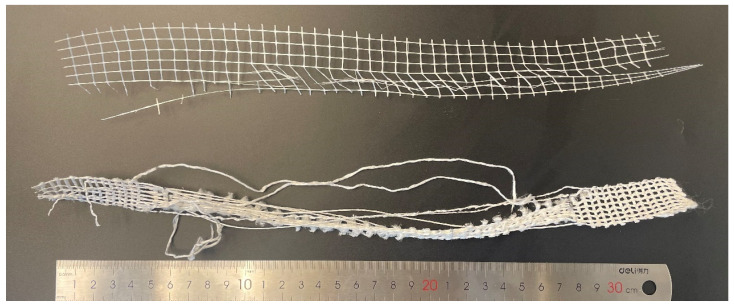
The failure pattern of fabrics.

The second reinforcement form tested was mesh fabric, divided into glass and UHMWPE. The grid size of the glass mesh fabric is 5 mm × 5 mm, as shown in Figure 2a. The structure of the UHMWPE fabric has an open-grid pattern with 2 mm × 2 mm rectangular cells. The warp consists of two twisted yarns, while the weft consists of one-way straight yarns that pass through the warp, as shown in Figure 2b. Their material properties are shown in Table 3, which were obtained from a uniaxial tensile test based on ASTM D882 and are consistent with the results of other studies [48,49]. The UHMWPE mesh fabric has higher tensile strength, greater Modulus, and greater ductility. Figure A1 and Figure A2 demonstrate the test setup and the failure pattern of the fabric. The mortar cover over the fabric was 5 mm from the bottom surface to ensure proper embedding of the fabric into the cementitious material [29,50,51].

**Figure 3:**
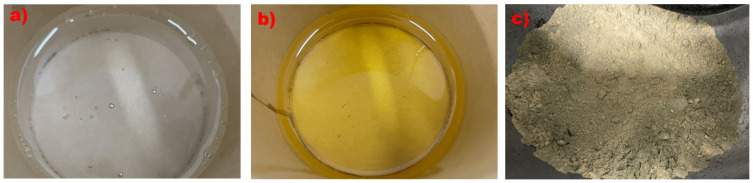
The two types of adhesives: (**a**) epoxy resin, (**b**) hardening agent, and (**c**) MPC.

The third method involved gluing UHMWPE fabric onto the UHPC surface using adhesive materials. The proper selection of bonding materials is crucial for enhancing the mechanical properties of the structural system, which consists of an FRP and a concrete matrix. According to ACI 440.2R, we tested two different adhesives to determine which provided the best bonding performance in this experiment using the wet layup FRP method. The adhesives evaluated were epoxy resin and MPC, as shown in Figure 3. The mechanical properties of these materials are shown in Table 4, with data provided by the manufacturers. According to the recommendations of the manufacturers, the mixing ratio for epoxy resin and its hardening agent is 2:1, while the ratio of magnesium oxide to phosphate in MPC is 1:1. The water-to-cement ratio for MPC is 0.16.

### 2.2. Experimental Setup

**Figure 4:**
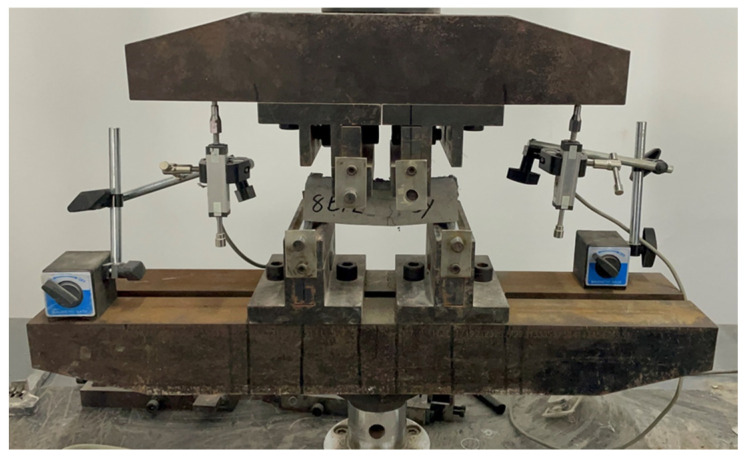
A four-point bending test for the UHPC specimen.

A four-point bending test was used to determine the mechanical behavior of each strengthened UHPC specimen, following ASTM D6272, as shown in Figure 4. The loading rate was 1 mm/min. Before automatic loading, a pre-load of approximately 60–80 N was applied to the specimen for consolidation. Two displacement gauges were placed on both sides, and real-time force–displacement curves were recorded. All of the experimental tests were carried out at Shike New Fiber Material Preparation Engineering (Technology) Center, Ningbo, China.

The experimental specimens were mainly divided into two material names, HSM and UHPC. HSM was a plain cementitious composite without reinforcement, while UHPC was HSM reinforced with fiber [29]. According to the recommendations of the Chinese code DBJ61/T112-2016, each experimental group has three identical HSM or UHPC specimens with dimensions of 160 mm × 40 mm × 40 mm. Specimens were cured at room temperature with relatively high moisture until they reached an age of 28 days.

A total of 45 specimens were prepared and tested, including HSM or UHPC reinforced with steel fibers, UHMWPE fibers, embedded UHMWPE fabric, and externally bonded FRPs. To ensure a clear understanding of the experimental results, each group of specimens was assigned a specific label. HSM represented plain specimens without any reinforcement. HSM_1PEI indicated specimens embedded with one layer of UHMWPE fabric. HSM_1GI indicated specimens embedded with one layer of glass fabric. HSM_1PEE_ER described specimens externally bonded with UHMWPE fabric using epoxy resin, and HSM_1PEE_MPC denoted those bonded with UHMWPE fabric using MPC. HSM_2PEE_ER referred to specimens externally bonded with two layers of UHMWPE fabric using epoxy resin. HSM_1GE indicated specimens externally bonded with one layer of glass fiber-reinforced polymers (GFRPs). UHPC_PE denoted specimens reinforced with UHMWPE fibers, while UHPC_S represented those reinforced with steel fibers. U_S_1PEE was used for steel fiber-reinforced UHPC externally bonded with one layer of UHMWPE FRPs, while U_S_1PEI represented steel fiber-reinforced UHPC internally embedded with one layer of UHMWPE FRPs. U_S_2PEI denoted steel fiber-reinforced UHPC embedded with two layers of UHMWPE fabric, and U_S_2PEE described steel fiber-reinforced UHPC externally bonded with two layers of UHMWPE fabric. U_PE_2PEI indicated UHMWPE fiber-reinforced UHPC externally bonded with one layer of UHMWPE fabric, and U_PE_2PEE described UHMWPE fiber-reinforced UHPC externally bonded with two layers of UHMWPE fabric. These designations are summarized in Table A1.

Combined with the recommendations of material manufacturers, the details of FRP installation on the specimen surface are as follows (taking epoxy resin as an example).

In the first step, the epoxy resin was cooled, and the surface of the concrete specimen was cleaned using abrasive paper and a rag. The epoxy resin and hardening agent were mixed at a ratio of 2:1. After 1–2 min of thorough mixing, the adhesive was applied to the first layer of the concrete surface with a thicker coat. A spiked roller was then utilized to press the fabric firmly during the pasting process. Following this pressing, a second coat of adhesive was evenly applied, and the spiked roller was used to press back and forth. A hardening time of 24–48 h was required for the epoxy resin.

**Figure 5:**
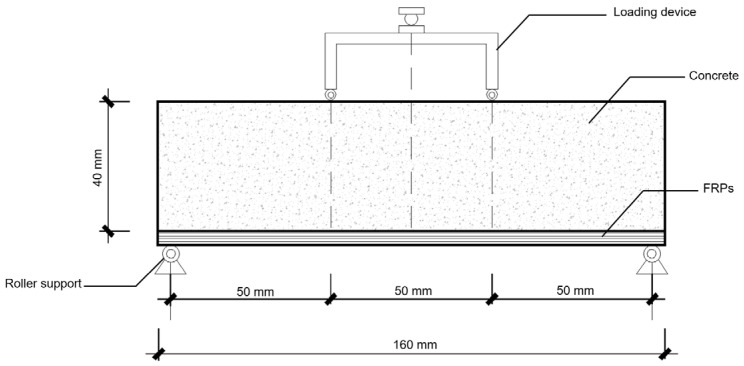
Schematic diagram of the four-point bending test for the concrete specimen wrapped with FRPs.

Once the bonding materials had hardened, the specimens with FRPs were placed on the four-point bending device. The FRP surface was positioned at the bottom to best resist flexural tension. The two loading points were applied at one-third of the length of the specimens, as shown in Figure 5.

Therefore, the flexural behavior of HSM or UHPC reinforced with different fibers or various fabric strengthening technologies can be compared by analyzing fracture mode, ductility, flexural strength, residual strength, and toughness.

## 3. Results and Discussion

### 3.1. Load–Displacement Curves

The load–displacement (L-D) curves of different HSM/UHPC specimens are shown in this section. The value of the *y*-axis represents the load applied through the hydraulic ram, while the displacement on the *x*-axis is the average of the values from two displacement gauges.

**Figure 6:**
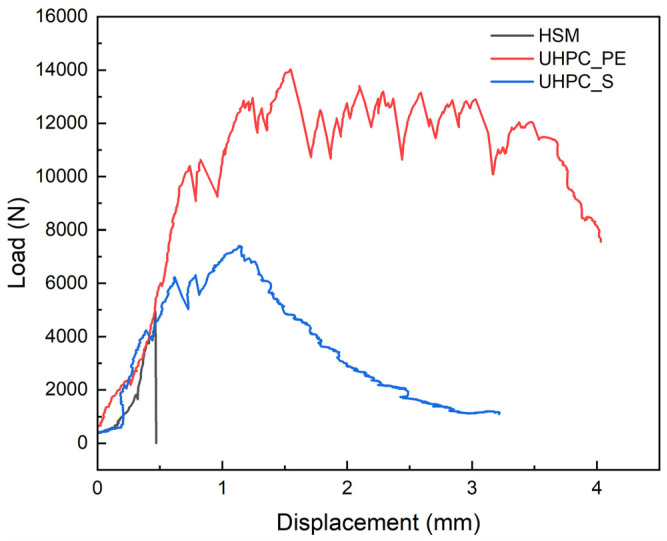
Comparison of load–displacement curves for specimens reinforced with different fibers.

The plain HSM is brittle, but different reinforcing materials and technologies can improve its bending properties. Effective reinforcing methods can not only resist cracking but also increase toughness. Incorporating fibers into the concrete matrix not only increases the elastic limit but also results in a pronounced post-cracking ductile behavior. Compared to steel fiber, UHMWPE fiber-reinforced UHPC exhibited superior strain-hardening and strain-softening performance, as shown in Figure 6. This strong and smooth strain-hardening behavior is due to the better ability of UHMWPE fibers to stretch and bridge cracks, which enhances energy absorption and ductility. The variable in Figure 6 is different fiber types. The first advantage of using UHMWPE reinforcement, incorporated in the form of fibers, is its better crack resistance and increased toughness.

**Figure 7:**
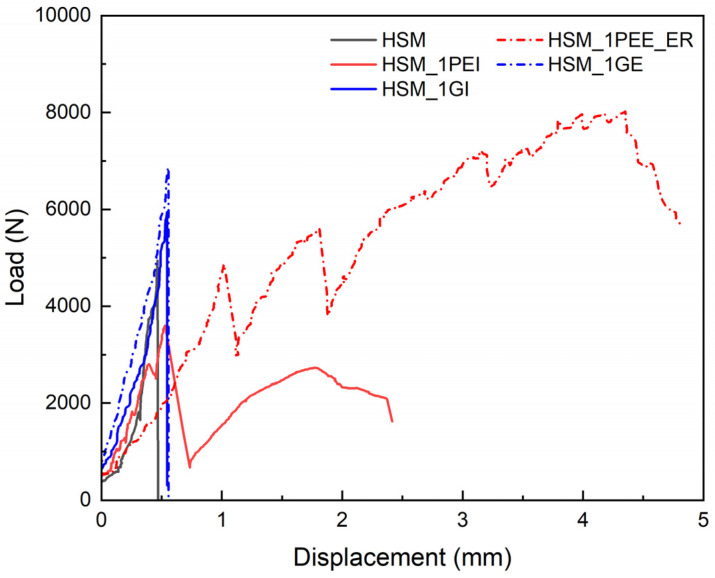
Comparison of load–displacement curves for specimens reinforced with different fabric materials.

The second reinforcement material is in the form of fabric. Figure 7 presents the L-D curves for the HSM matrix strengthened either internally or externally using different fabric materials, including glass and UHMWPE. The solid black curve represents plain HSM without any reinforcement for comparison. The other solid curves show fabrics applied internally. The short dash–dot curves represent fabrics bonded externally. The red curve corresponds to UHMWPE, and the blue curve corresponds to glass. Using UHMWPE significantly improves the bending ductility of specimens, resulting in greater energy absorption compared to glass fabric. Meanwhile, reinforcement with glass fabric could significantly increase the load at cracking, but then it failed suddenly. The strengthening effect of using fabric to reinforce the matrix externally was better than that of internal reinforcement.

**Figure 8:**
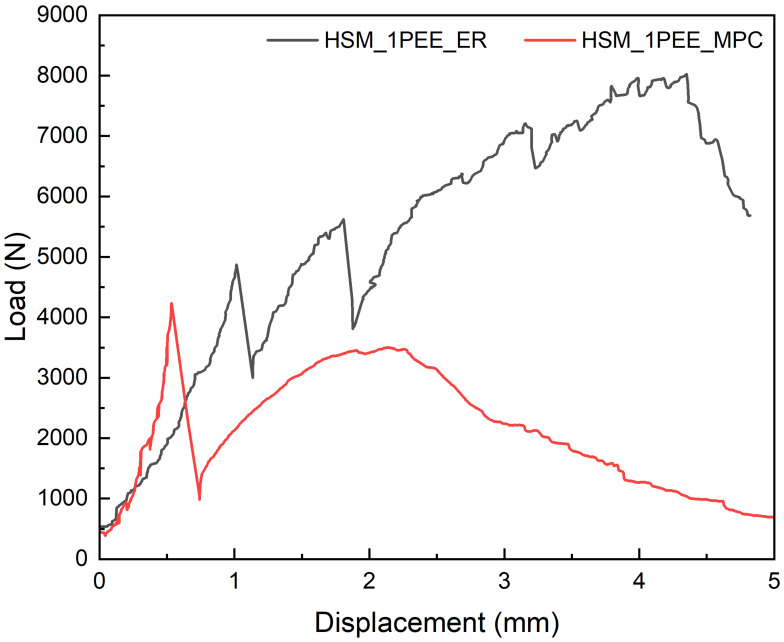
Comparison of load–displacement curves for specimens externally bonded with UHMWPE fabric using different adhesives.

When it comes to HSM externally bonded with UHMWPE fabric, using different adhesives was investigated. Figure 8 demonstrates the L-D curves of specimens externally bonded with UHMWPE fabric using epoxy resin and MPC. It clearly illustrates that the flexural improvement in specimens using epoxy resin was overwhelmingly higher than when using MPC, including in cracking strength, ultimate strength, ductility, and toughness.

**Figure 9:**
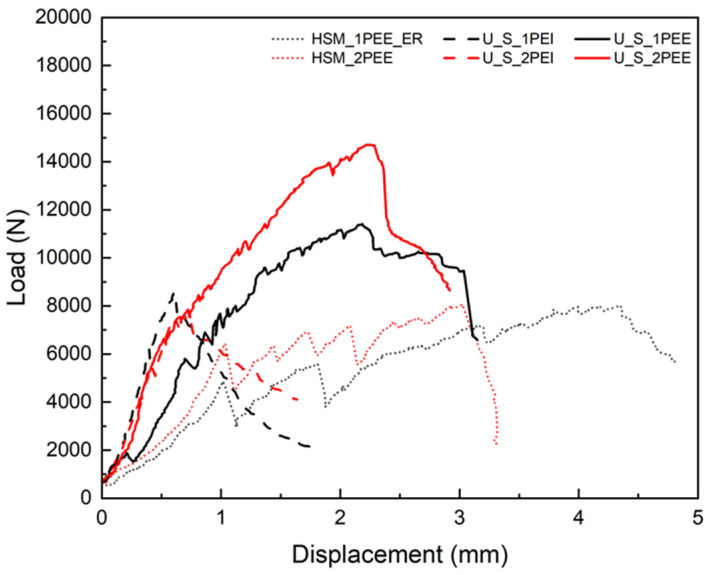
Comparison of load–displacement curves for specimens reinforced with different numbers of UHMWPE fabric layers.

The layer number of applying UHMWPE fabrics is also a basic and significant parameter to consider for reinforcing external or internal specimens, as shown in Figure 9. The black line is applied to one-layer UHMWPE fabric, and the red one is applied to two layers. The short dot curves are for HSM strengthened with fabric externally, the dash curves are for steel fiber-reinforced UHPC strengthened with fabric internally, and the solid curves are for steel fiber-reinforced UHPC strengthened with fabric externally. Both matrix type and strengthening techniques affect the impact of layer number on the flexural performance of the specimens. It can be observed that the stronger the initial reinforcement, the greater the improvement when the number of fabric layers is increased. The mechanical behavior of steel fiber-reinforced UHPC was overwhelmingly better than that of HSM, and external UHMWPE reinforcement proved to be a more effective strengthening technique than internal reinforcement, as shown in Figure 6 and Figure 7.

**Figure 10:**
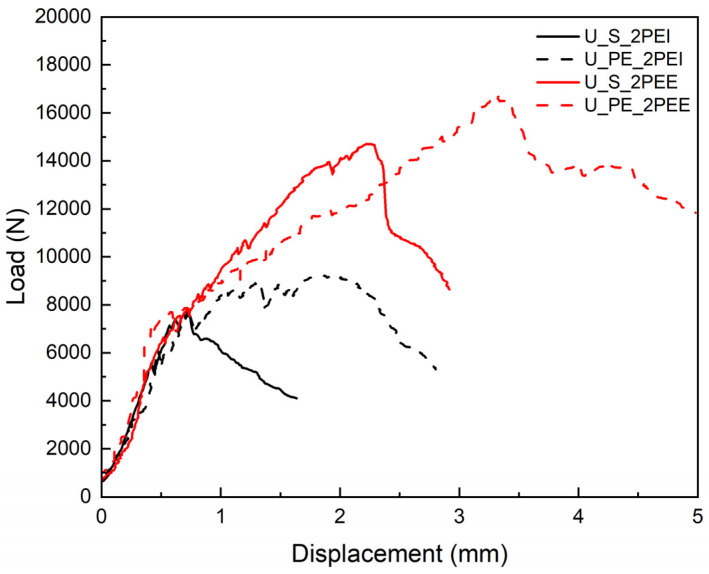
Comparison of load–displacement curves for specimens reinforced with different combined strengthening techniques.

Therefore, the combined strengthening techniques should be investigated for improved flexural behavior, as shown in Figure 10. Due to the higher flexural performance of UHPC_PE, steel fiber-reinforced UHPC can be replaced with UHMWPE. Using UHMWPE material achieves higher bending strength, ductility, and toughness for both internal and external reinforcement.

Through the comparison of different L-D curves, several effective UHMWPE-based material-strengthening techniques can be identified. It is valuable to quantify the improvements in flexural strength, ductility, and toughness of these reinforcement methods for comparative analysis.

### 3.2. Flexural Properties

**Figure 11:**
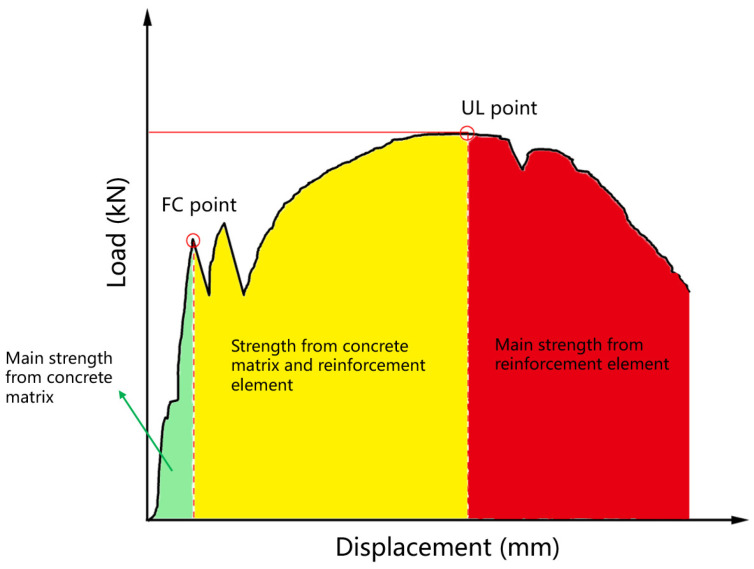
Schematic diagram of a typical load–displacement curve for reinforced UHPC.

To better compare the flexural properties of each experimental group, the data from the L-D curves require a more thorough theoretical analysis. As early as 1987, Mangat and Gurusamy [52] summarized existing theories on the changes in the flexural strength of fiber-reinforced beams, emphasizing the importance of appropriate L-D curves. Two critical points on the typical L-D curve should be noted: the first crack (FC) point and the ultimate load (UL) point. Meng et al. [29] also used the corresponding load and displacement at these two points. The load and displacement at the FC point and the UL point are commonly used to evaluate the bearing capacity of the bending specimen [53,54,55]. ASTM C1609 defined the FC point as the first peak where the slope of the curve changes from a steep rise to a smooth or slight decline, that is, the point when the slope is zero for the first time [56,57]. Until this point, the flexural behavior is primarily determined by the strength of the concrete matrix, represented by the green area in Figure 11. The UL point is identified as the peak of the curve during the hardening phase [58,59,60]. As cracks form and propagate in the concrete surface, the reinforcing elements begin to share the load, as depicted in the yellow area. After the UL point, the mechanical capacity of the matrix would fail, and the flexural strength depends mainly on the reinforcing materials, represented by the red area.

Therefore, the load ($P_{FC}$), the displacement ($\delta_{FC}$), and the bending stress ($f_{FC}$) at the FC point and those at the UL point for each experimental group are compared, as shown in Table 5. The values in Table 5 are the average of three specimens in the test results. According to ASTM D6272, the bending stress was calculated using the following equation:

(1) $$\sigma_{b}=PL/bd^{2}$$

In Equation (1), $\sigma_{b}$ is the bending stress in MPa. P is the load at a given point on the L-D curve in N. L is the support span in mm. b is the width of the beam in mm. d is the depth of the beam in mm.

In Table 5 above, the specimen names were first divided into three main categories based on whether they incorporated steel fiber, UHMWPE, or neither. This can be easily recognized from the first part of the specimen names, such as HSM, U_S, and U_PE. The second part of the names represents the locations where mesh fabrics were strengthened or the types of fabric used. For example, 2PEI indicates the embedding of two layers of UHMWPE mesh fabrics internally, while 1GE denotes the external bonding of glass mesh fabric.

The data for these 15 experimental groups represent the averages of the results. Each group contained three specimens, totaling forty-five specimens that were investigated and compared. The effects of fabric layer, fiber type, fabric type, adhesive type, and various combined strengthening techniques on the improvement of flexural performance were considered.

Due to the absence of fiber or fabric reinforcement, HSM exhibited a weak plastic deformation capacity. Even though one layer of glass mesh fabric was bonded internally or externally, the specimens still demonstrated brittle bending behavior, with final failure occurring suddenly at the FC point. Although the tensile properties of UHMWPE fabric were higher than those of glass fabric, internally bonding UHMWPE mesh fabric did not improve the bending strength or ductility of HSM. When UHMWPE fabric was externally bonded using MPC, the FC point occurred earlier, and there was no obvious improvement in flexural behavior.

In the group of UHPC reinforced with steel fiber, bonding UHMWPE fabrics either internally or externally resulted in higher bending strength at both the FC and UL points. Externally wrapping with UHMWPE fabrics also led to larger deformation.

For the UHPC groups incorporating UHMWPE fiber, the specimens exhibited significantly better mechanical strength and ductility than the previous groups. Therefore, a thorough and transparent analysis of load capacity and ductility is necessary to compare and discuss the flexural reinforcement effects of UHMWPE material in the HSM/UHPC matrix.

### 3.3. Load-Carrying Capacity—Strength

The bending strength is calculated in Table 5. The bending stress at the FC point represents the strength of the matrix [61], while the stress at the UL point represents the strength of the reinforcement matrix adhesive composite [62].

**Figure 12:**
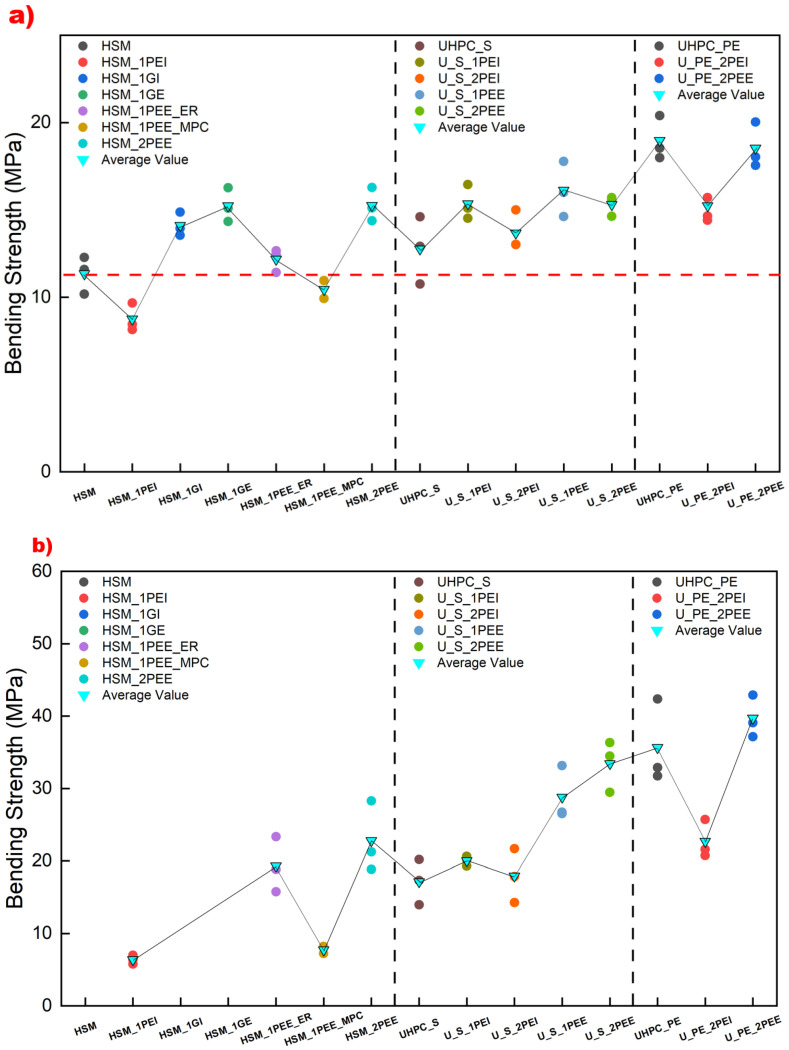
Comparative diagram of bending strength in experimental specimens at (**a**) the FC point and (**b**) the UL point.

Figure 12 shows the comparative results of bending stress at the FC point and the UL point for each specimen. Each colorful ball represents a test specimen result, while the blue reverse triangle indicates the average value for each experimental group. The horizontal red dashed line in Figure 12a represents the failure data of HSM, showing that most reinforcing methods can improve cracking strength. The intermediate vertical dashed line divides the specimen results into three groups: unreinforced fibers, steel fiber reinforcement, and UHMWPE fiber reinforcement, respectively. The UHMWPE fiber-reinforced UHPC groups exhibit higher cracking and ultimate strength than the others. Embedding UHMWPE fabric internally or externally bonding it using MPC can induce earlier cracking in HSM and UHPC reinforced with steel fiber, but embedding UHMWPE fabric internally in steel fiber-reinforced UHPC can improve bending strength at both the FC and UL points. HSM, HSM_1GI, and HSM_1GE did not exhibit any post-cracking behavior, so their ultimate strength at the UL point was negligible, as shown in Figure 12b. Increasing the number of fabric layers is beneficial for the ultimate strength of steel-reinforced UHPC externally bonded with fabric. Externally bonding UHMWPE fabric using epoxy resin consistently proved to be the most effective technique for improving ultimate strength. Among all the strengthening techniques, U_PE_2PEE exhibited the highest bending strength, around 40 MPa, compared to the failure value of HSM, which was only 11.3 MPa, demonstrating the superiority of UHMWPE-based material for flexural reinforcement in UHPC.

### 3.4. Ductility—Displacement

**Figure 13:**
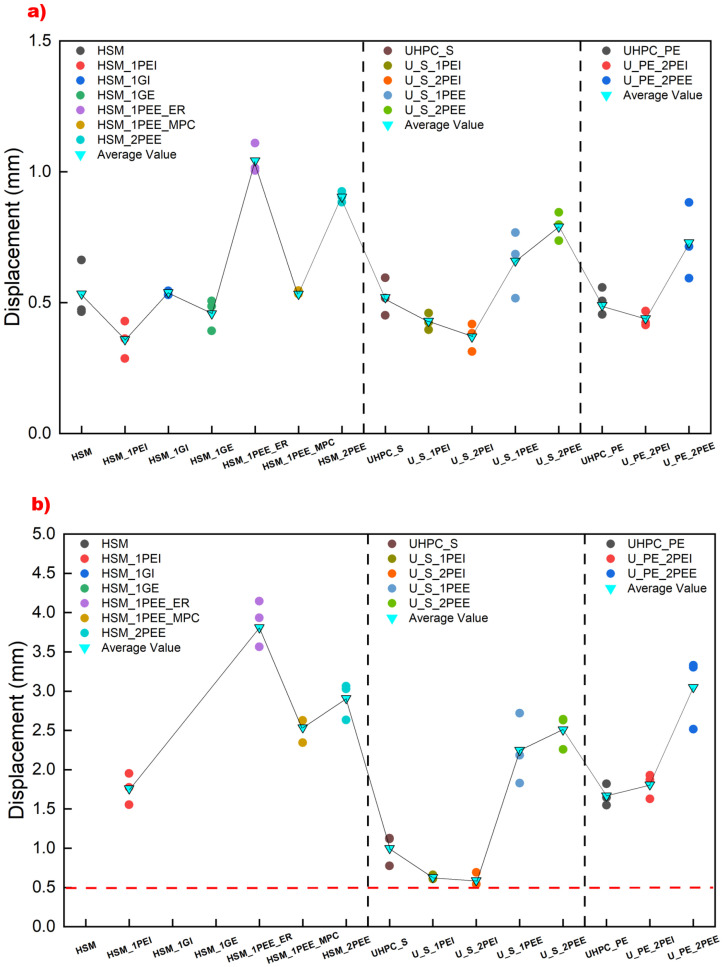
Comparative diagram of displacement in experimental specimens at (**a**) the FC point and (**b**) the UL point.

The displacement value is a crucial parameter for assessing the ductility of a material or structure [63,64,65], and the effect of each strengthening technique on displacement at the FC point and UL point is shown in Figure 13. In Figure 13a, the brittleness of unreinforced HSM is illustrated by a displacement of around 0.5 mm at mechanical failure. Incorporating fiber failed to delay crack formation but improved the ductility in the post-cracking phase, as indicated by the horizontal dashed red line in Figure 13b, which represents the failure displacement of unreinforced HSM. However, in every group, including HSM, UHPC_S, and UHPC_PE, it is observed that externally bonding UHMWPE fabric using epoxy resin significantly improves the ductile behavior of the specimens, not only in the cracking phase but also in the post-cracking phase. This demonstrates that this strengthening technique greatly enhances the deformation performance of UHMWPE.

### 3.5. Energy Absorption Capacity—Toughness

**Figure 14:**
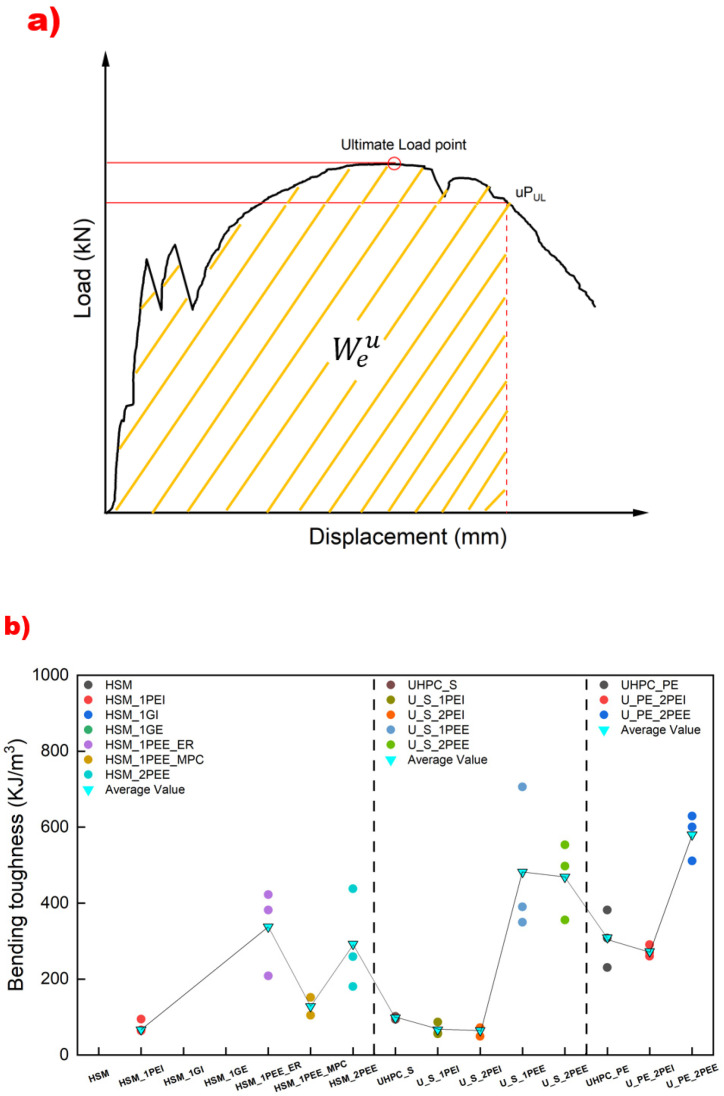
(**a**) Schematic diagram for calculating bending toughness and (**b**) comparative diagram of bending toughness in experimental specimens.

Toughness is measured by calculating the area under the L-D curve to a certain value [66,67,68], which is a more important and comprehensive parameter to evaluate the flexural behavior of a structure, as shown in Figure 14a. This method has also been adopted in recent studies on sustainable reinforcement techniques, including the use of recycled plastic mesh to enhance flexural performance in concrete and mortar beams [69,70]. The bending toughness was calculated using the following equations:

(2) $$f_{eq}^{u}=\frac{\Omega_{u}L}{bh^{2}\delta_{u}}$$

(3) $$W_{e}^{u}=\frac{\Omega_{u}}{bh^{2}}$$

In Equations (2) and (3), $f_{eq}^{u}$ is the equivalent bending strength in MPa. $\Omega_{u}$ is the area under the L-D curve when the displacement is $\delta_{u}$ in N·mm. $\delta_{u}$ is the corresponding displacement value when the load drops to u times the last peak load in mm. u can be 0.85, 0.50, and 0.20. L is the supporting span in mm. b is the width of the beam in mm. h is the depth of the beam in mm. $W_{e}^{u}$ is the equivalent bending toughness in KJ/m^3^.

Figure 14b illustrates the comparative results for bending toughness across different experimental groups. The unreinforced HSM, HSM_1GI, and HSM_1GE failed suddenly once the crack formed, so their toughness values were negligible. The results of the toughness comparison were mainly influenced by the ductile properties of the strengthening techniques, like Figure 14b. Compared to glass mesh fabric reinforcement, using UHMWPE fabric improved toughness. The toughness values of HSM_1PEI and HSM_1PEE were 67 KJ/m^3^ and 337 KJ/m^3^, respectively, both significantly higher than the null values of HSM, HSM_1GI, and HSM_1GE. Using epoxy resin for external bonding was far more effective than using MPC, with their respective values being 337 KJ/m^3^ and 128 KJ/m^3^. The number of fabric layers did not affect toughness, as shown by similar results for HSM_1PEE_ER and HSM_2PEE, U_S_1PEI and U_S_2PEI, and U_S_1PEE and U_S_2PEE. Incorporating UHMWPE fiber greatly improved toughness performance, with UHPC_PE achieving 309 KJ/m^3^, significantly higher than UHPC_S, which achieved 98 KJ/m^3^. Additionally, internal reinforcement with UHMWPE fabric was not as effective as external bonding, as seen in the comparisons between U_S_1PEI (66.8 KJ/m^3^) and U_S_1PEE (481.9 KJ/m^3^), U_S_2PEI (64.6 KJ/m^3^) and U_S_2PEE (468.8 KJ/m^3^), and U_PE_2PEI (273.6 KJ/m^3^) and U_PE_2PEE (580.2 KJ/m^3^), in terms of flexural toughness.

To enhance the transparency of the results and provide statistical context, the mean and standard deviation of toughness values from three replicates in each group are summarized in Table 6. This table serves to demonstrate the consistency and reliability of the test data.

### 3.6. Cost–Performance Analysis

To assess the economic feasibility of the proposed UHMWPE-based reinforcement methods, a simple cost–performance analysis was conducted. The comparison is based on the unit material cost per cubic meter of UHPC and the corresponding average flexural toughness achieved from the experiments, as shown in Table 7.

Among all the tested specimens, the UHPC specimen reinforced with two layers of UHMWPE fabric (U_PE_2PEE) demonstrated the highest cost–performance, achieving a toughness of 580.2 KJ/m^3^ at a fabric cost of 82.8 USD/m^3^, resulting in 7.0 KJ/USD. In contrast, the steel fiber-reinforced UHPC (UHPC_S) exhibited much lower cost effectiveness with only 1.0 KJ/USD, despite its toughness of 98.1 KJ/m^3^, due to the high cost of steel fibers (95 USD/m^3^).

Overall, the data clearly show that UHMWPE fabric reinforcement, especially when externally bonded, provides a significantly more favorable balance between structural performance and material cost than traditional steel or internal fiber reinforcements.

### 3.7. Fracture Mechanism

**Figure A3:**
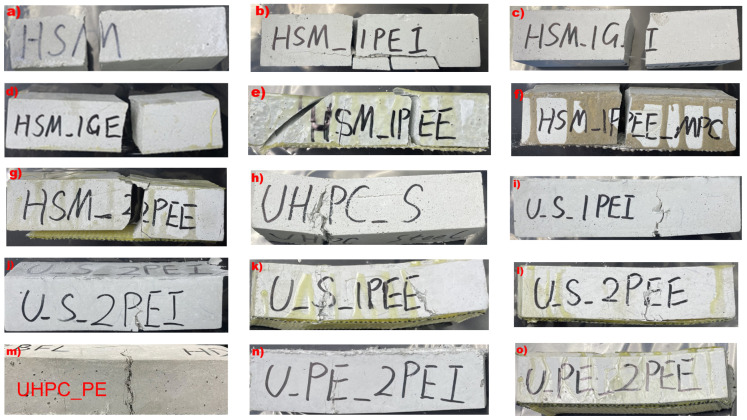
Fracture of each UHPC specimen: (**a**) HSM, (**b**) HSM_1PEI, (**c**) HSM_1GI, (**d**) HSM_1GE, (**e**) HSM_1PEE_ER, (**f**) HSM_1PEE_MPC, (**g**) HSM_2PEE, (**h**) UHPC_S, (**i**) U_S_1PEI, (**j**) U_S_2PEI, (**k**) U_S_1PEE, (**l**) U_S_2PEE, (**m**) UHPC_PE, (**n**) U_PE_2PEI, and (**o**) U_PE_2PEE.

Figure A3 demonstrates the different fracture modes for each UHPC specimen. The HSM, HSM_1GI, and HSM_1GE specimens failed suddenly with a loud noise, and the specimens split into two pieces, as shown in Figure A3a,c,d. For HSM_1PEI and HSM_1PEE, the UHMWPE fabric maintained the integrity of the matrix due to its greater strength, modulus, and elongation capacity. As shown in Figure A3b, the initial crack started at the bottom of the HSM, expanded vertically toward the UHMWPE fabric, and then split into two. One crack propagated horizontally along the fabric, while the other continued vertically into the matrix. In Figure A3e–g, the failure of the specimens was mainly caused by FRP debonding.

When fiber was incorporated into the matrix, the ductility and toughness were largely improved, which can be observed by the transition from a sudden rupture to a gradual cracking process. Especially, in Figure A3h,m, the formation of micro-cracks, rather than a single large crack, can be seen around the break area. This situation allows more fibers to engage and redistribute stress, leading to a multi-crack propagation pattern and resulting in superior strain-hardening and -softening behavior.

Some combined reinforcing schemes showed better improvement in mechanical performance. For example, adding fiber and externally bonding fabric resulted in great integrity and a multi-crack propagation pattern, as shown in Figure A3k,l,o, leading to higher bending strength and greater toughness. However, some fracture results were unexpected, such as internally bonding UHMWPE fabric. In the work of Meng et al. [29], they found a similar problem. It was explained by the fact that the mechanical strength of the matrix was lost before the mechanical properties of the UHMWPE fabric could be utilized. A synergistic effect between the concrete matrix and reinforcement element is significant, especially in the yellow area in Figure A3. Therefore, the epoxy resin–UHMWPE composite achieved a good synergistic effect with the concrete matrix.

The relatively poor performance of the internally embedded UHMWPE fabric is primarily due to the lack of effective interfacial bonding with the cement matrix. Unlike external reinforcement, where epoxy resin creates a strong bond between the fabric and the surface, the embedded fabric was simply surrounded by mortar without any adhesive enhancement. As shown in Table 4, epoxy resin has a much higher tensile and bending strength than magnesium phosphate cement, highlighting the importance of adhesive properties. In internal reinforcement, the fabric could not be fully activated due to premature failure of the brittle matrix, and much of the fabric’s tensile capacity was not utilized. In contrast, externally bonded UHMWPE fabric, anchored by epoxy resin, maintained better synergy with the matrix, allowing for greater deformation and improved post-crack flexural performance.

**Figure 15:**
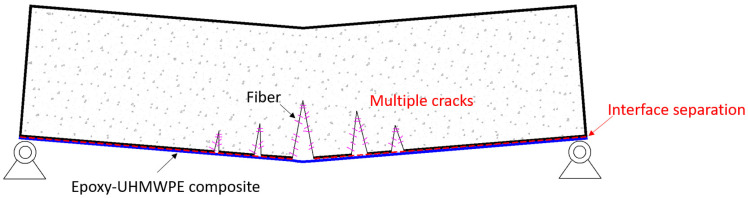
Schematic representation of the fracture mechanism of UHMWPE fabric externally bonded with epoxy resin to a cementitious matrix under flexural loading.

As illustrated in Figure 15, a schematic diagram of the fracture mechanism of the UHPC beam reinforced with the epoxy–UHMWPE composite is presented. Under flexural loading, the fiber-reinforced UHPC matrix initially develops micro-cracks, which gradually propagate and multiply due to the presence of distributed fibers. These fibers bridge the cracks and help delay their growth, resulting in a multi-crack propagation pattern rather than a single dominant fracture. This behavior enhances ductility and toughness.

The externally bonded UHMWPE fabric, anchored with epoxy resin, remains well-bonded to the matrix, providing effective confinement and tensile reinforcement. However, localized interface separation may still occur at the end due to high-stress concentration near the supports. Figure 15 highlights the synergistic action between the fiber, matrix, and epoxy–UHMWPE composite layer, demonstrating how the composite reinforcement contributes to better crack control, energy dissipation, and post-cracking performance.

## 4. Conclusions

This study explored the flexural behavior of UHPC reinforced with UHMWPE-based materials. A comprehensive assessment of various parameters was conducted, including the effects of fabric layers, fiber types, fabric types, adhesive types, and different combined strengthening techniques. Based on the test results, a systematic mechanical analysis was performed, covering strength, ductility, and toughness. Furthermore, the illustration of the fracture mechanism provides specific insights into the failure modes, aligning with the mechanical analysis. The main findings can be summarized as follows:

1. Incorporating UHMWPE fiber into the HSM matrix resulted in superior flexural performance compared to steel fiber. The average bending strength with UHMWPE fiber and steel fiber was 36 MPa and 17 MPa, respectively. Their average ductility was 1.7 mm and 1 mm, while their toughness was 98 KJ/m^3^ and 307 KJ/m^3^, respectively.
2. The UHMWPE mesh fabric has higher strength and greater elongation capacity than glass fabric. Therefore, reinforcing the matrix with both internally and externally bonded UHMWPE fabric can better accommodate matrix deformation, improving ductility and toughness and thereby maintaining structural integrity.
3. Compared to MPC, epoxy resin exhibited better material properties. As a result, epoxy resin adhered more effectively to UHMWPE fabric on the concrete surface. The epoxy resin–UHMWPE composite achieved a strong synergistic effect with the concrete matrix.
4. Applying more layers can improve the flexural performance of externally bonded UHMWPE fabric, but it does not benefit the strengthening technique when the fabric is embedded internally. Therefore, increasing the number of layers is only advantageous when the strengthening technique ensures good mechanical cooperation.

Based on the above findings, the best-combined strengthening technique in this study was the incorporation of UHMWPE fiber along with externally bonded UHMWPE fabric. The U_PE_2PEE specimen exhibited the highest ultimate bending strength and greatest toughness among all test groups, with values of 40 MPa and 580 KJ/m^3^, respectively. This is because the incorporation of UHMWPE fiber converts large cracks into multiple micro-cracks, allowing the fiber to redistribute stress more effectively than steel fiber. Additionally, the external UHMWPE fabric coordinated well with the internal UHMWPE fiber during deformation, resulting in greater structural integrity and reducing the issue of FRP debonding.

## 5. Limitations and Future Work

Although this study provides a detailed experimental investigation into the short-term flexural performance of UHMWPE-reinforced UHPC, several limitations should be acknowledged. First, the present work did not include long-term durability tests, such as water absorption, freeze–thaw cycles, or chemical resistance, which are essential for evaluating the performance of the material under actual environmental conditions.

Second, the microscopic interaction and bond development between the UHMWPE fibers/fabrics and the cementitious matrix were not investigated. In particular, the surface morphology and chemical characteristics of the reinforcement materials can significantly influence the interfacial bonding strength, thereby affecting crack propagation modes and overall energy dissipation. For example, rough or chemically treated surfaces may enhance mechanical interlocking and bond strength, while smoother or inert surfaces can lead to weaker adhesion and more frequent fiber pullout.

Moreover, the long-term effects of environmental factors such as UV exposure, moisture, and temperature fluctuations on UHMWPE’s mechanical performance have not yet been explored and should be included in future durability evaluations.

Although UHMWPE fibers and fabrics demonstrated ease of handling and safety advantages in laboratory settings, further investigation is required to assess their applicability and constructability under real-world site conditions.

Future research should consider incorporating durability testing under various environmental exposures to assess the long-term service life and reliability of UHMWPE-based reinforcement, particularly under UV radiation, temperature cycling, and humid conditions. In addition, microscopic and chemical analyses such as SEM, EDS, and FTIR are recommended to explore the interfacial bonding mechanism in more depth. Further studies on aging effects, fatigue performance under repeated loading, and field-scale constructability are also essential to support the broader application of UHMWPE in real-world concrete structures.

## Figures and Tables

**Table 1:** Experimental proportions of UHPC mixtures.

Ingredient	Quantity (kg/t)	Ingredient	Quantity (kg/t)
525 Cement	350	Medium sand (0.35–0.5 mm)	350
Fly ash	100	Find sand (0.25–0.35 mm)	75
Silica fume	50	Superplasticizer	2.5
Coarse sand (>0.5 mm)	75	Water–cement ratio	0.17

**Table 2:** Characteristics of fiber-reinforcing materials.

Fiber Type	Length (mm)	Aspect Ratio	Density (kg/m^3^)	Tensile Strength (MPa)	Elastic Modulus (GPa)
Steel	13	65	7900	2850	250
UHMWPE	12	85	900	2000	105

**Table 3:** Characteristics of textile-reinforcing materials.

Fabric Material	Tensile Strength (MPa)	Young’s Modulus (GPa)	Elongation at Break (%)	Thickness (mm)
Glass	301	8.99	3.4%	0.3
UHMWPE	1432	12.36	7.0%	0.8

The data in this table are the average values of 5 specimens in each group.

**Table 4:** Characteristics of adhesive materials.

Adhesive Type	Compressive Strength (MPa)	Tensile Strength (MPa)	Tensile Modulus (GPa)	Bending Strength (MPa)
Epoxy resin	70	40	2.5	50
MPC	56.3	6.5	2	8.1

**Table 5:** Flexural properties of each experimental group at two key points.

Specimen Name	Key Points					
	FC Point			UL Point (Post-Cracking Phase)		
	$P_{FC}$ (N)	$\delta_{FC}$ (mm)	$f_{FC}$ (MPa)	$P_{UL}$ (N)	$\delta_{UL}$ (mm)	$f_{UL}$ (MPa)
HSM	4840.50	0.53	11.35	-	-	-
HSM_1PEI	3733.26	0.36	8.75	2727.71	1.76	6.40
HSM_1GI	6023.55	0.54	14.12	-	-	-
HSM_1GE	6499.11	0.46	15.23	-	-	-
HSM_1PEE_ER	5193.53	1.04	12.17	8244.23	3.81	19.32
HSM_1PEE_MPC	2337.13	0.36	5.48	4454.48	2.54	10.44
HSM_2PEE	6510.27	0.90	15.26	9723.18	2.91	22.79
UHPC_S	5444.43	0.52	12.76	7320.74	1.00	17.16
U_S_1PEI	6551.91	0.43	15.36	8548.61	0.63	20.04
U_S_2PEI	5835.95	0.37	13.68	7650.87	0.59	17.93
U_S_1PEE	6883.92	0.66	16.13	12,287.65	2.25	28.80
U_S_2PEE	6530.47	0.79	15.31	14,263.28	2.51	33.43
UHPC_PE	8096.46	0.49	18.98	14,029.9	1.67	35.66
U_PE_2PEI	6366.69	0.44	14.92	9677.88	1.81	22.68
U_PE_2PEE	7908.02	0.73	18.53	16,941.94	3.05	39.71

**Table 6:** Summary of the mean and standard deviation for each specimen group.

Specimen Name	Key Points					
	Strength				Displacement	
	FC Point		UL Point		FC Point	
	Mean (MPa)	Standard Deviation (MPa)	Mean (MPa)	Standard Deviation (N)	Mean (mm)	Standard Deviation (mm)
HSM	11.34	1.07	-	-	0.53	0.11
HSM_1PEI	8.75	0.81	6.39	0.61	0.36	0.15
HSM_1GI	14.12	0.68	-	-	0.54	0.01
HSM_1GE	15.2	2.58	-	-	0.46	0.14
HSM_1PEE_ER	12.17	0.66	19.32	3.81	1.04	0.06
HSM_1PEE_MPC	10.44	0.73	7.73	0.69	0.54	0.01
HSM_2PEE	15.26	0.96	22.79	4.91	1.06	0.04
UHPC_S	17.16	3.11	17.16	3.11	0.66	0.05
U_S_1PEI	15.36	1.00	20.04	0.66	0.43	0.03
U_S_2PEI	13.68	1.14	17.93	3.70	0.37	0.05
U_S_1PEE	15.36	1.00	28.80	3.76	0.66	0.13
U_S_2PEE	13.68	1.14	33.43	3.55	0.61	0.13
UHPC_PE	35.66	5.84	35.66	5.84	0.46	0.02
U_PE_2PEI	14.92	0.69	22.68	2.66	0.44	0.03
U_PE_2PEE	18.53	2.28	39.71	2.93	0.73	0.15
**Specimen Name**	**Displacement**		**Toughness**			
	**UL Point**					
	**Mean** **(** **mm)**	**Standard Deviation** **(** **mm)**	**Mean** **(** **KJ/m^3^)**	**Standard Deviation** **(KJ/m^3^)**		
HSM	-	-	-	-		
HSM_1PEI	1.76	0.20	75.05	17.16		
HSM_1GI	-	-	-	-		
HSM_1GE	-	-	-	-		
HSM_1PEE_ER	3.81	0.60	337.47	113.81		
HSM_1PEE_MPC	2.54	0.55	128.24	33.31		
HSM_2PEE	3.24	0.74	292.43	131.94		
UHPC_S	1.01	0.20	98.11	3.64		
U_S_1PEI	0.63	0.03	66.82	17.18		
U_S_2PEI	0.59	0.09	64.60	13.18		
U_S_1PEE	2.25	0.45	481.90	195.10		
U_S_2PEE	2.51	0.22	468.82	102.07		
UHPC_PE	1.68	0.19	306.80	75.80		
U_PE_2PEI	1.54	0.02	273.58	15.35		
U_PE_2PEE	3.05	0.46	580.19	61.41		

**Table 7:** Cost–performance comparison.

Specimen Name	Toughness (KJ/m^3^)	Estimated Fiber/Fabric Cost (USD/m^3^)	Toughness per Dollar (KJ/USD)
HSM	-	-	-
HSM_1PEI	75.05	21.4	3.51
HSM_1GI	-	1.69	-
HSM_1GE	-	1.69	-
HSM_1PEE_ER	337.5	21.4	15.77
HSM_1PEE_MPC	128.2	21.4	5.99
HSM_2PEE	292.4	42.8	6.83
UHPC_S	98.1	95	1.0
U_S_1PEI	66.8	116.4	0.57
U_S_2PEI	64.6	137.8	0.47
U_S_1PEE	481.9	116.4	4.14
U_S_2PEE	468.8	137.8	3.40
UHPC_PE	306.8	40	7.67
U_PE_2PEI	273.58	82.8	3.30
U_PE_2PEE	580.2	82.8	7.0

Cost estimates are based on material prices and experimented dosages. Exact values may vary by regional supplier and concrete size.

## Data Availability

The original contributions presented in the study are included in the article; further inquiries can be directed to the corresponding author.

## Abbreviations

Abbreviation	Full Form
UHPC	Ultra-High-Performance Concrete
UHWMPE	Ultra-High Molecular Weight Polyethylene
FRP	Fiber-Reinforced Polymer
HSM	High-Strength Mortar
MPC	Magnesium Phosphate Cement

## Appendix A

**Table A1:** The designation of the experimental specimen groups.

Group Name	Schematic Diagram
HSM (reference)	
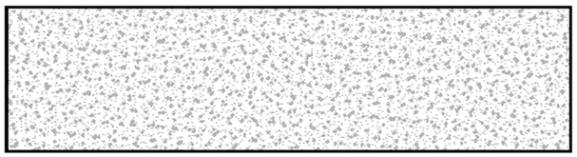
UHPC_PE	
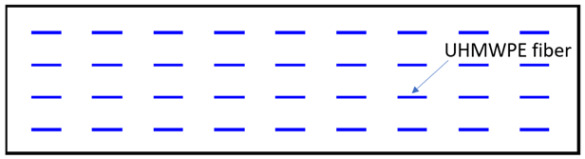
UHPC_S	
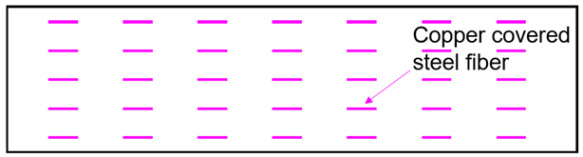
HSM_1PEI	
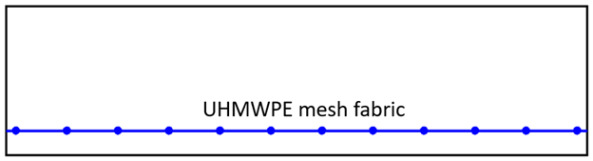
HSM_1GI	
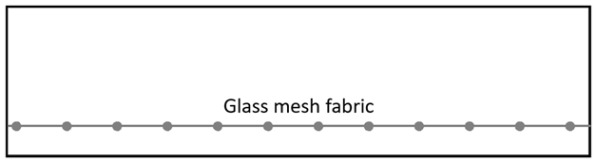
HSM_1PEE_ER	
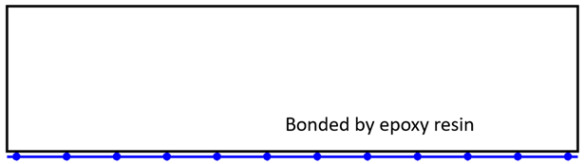
HSM_1PEE_MPC	
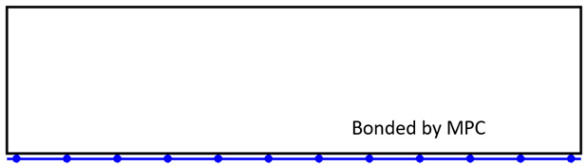
HSM_2PEE_ER (the subsequent groups all used ER)	
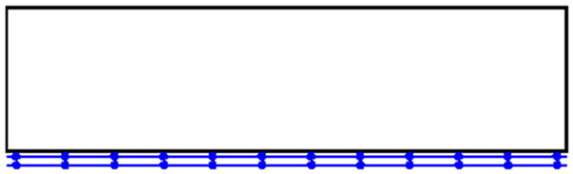
HSM_1GE	
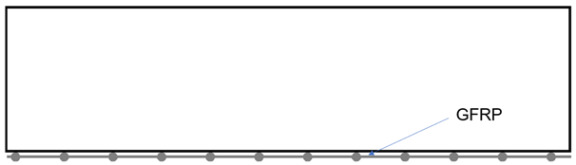
U_S_1PEE	
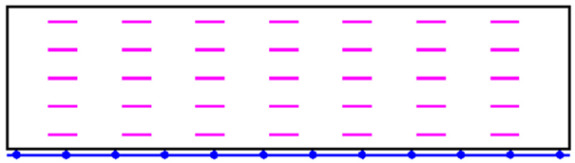
U_S_1PEI	
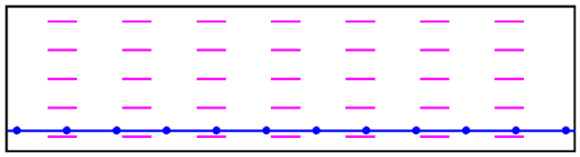
U_S_2PEI	
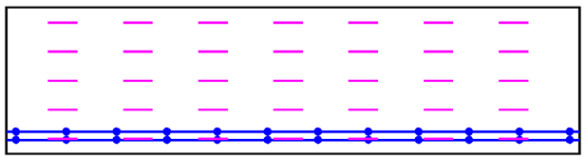
U_S_2PEE	
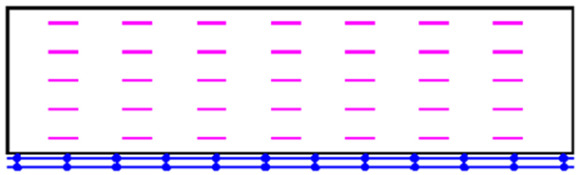
U_PE_2PEI	
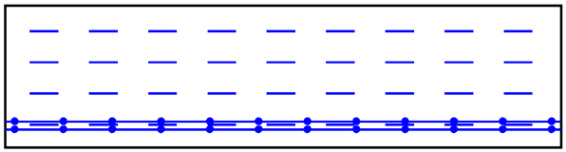
U_PE_2PEE	
